# *Brachybacterium epidermidis* Sp. Nov., a Novel Bacterial Species Isolated from the Back of the Right Hand, in a 67-Year-Old Healthy Woman

**DOI:** 10.1155/2022/2875994

**Published:** 2022-03-29

**Authors:** Manon Boxberger, Sibylle Magnien, Angéline Antezack, Clara Rolland, Marine Makoa, Bernard La-Scola, Nadim Cassir

**Affiliations:** ^1^IHU Méditerranée Infection, Marseille, France; ^2^Institut de Recherche Pour le Développement (IRD), Assistance Publique-Hôpitaux de Marseille (AP-HM), Microbes,Evolution,Phylogénie et Infection (MEPHI), Aix-Marseille Université, Marseille, France

## Abstract

Knowledge on human skin microbiota composition has been expanding in recent years. Its role in human health and disease represents an active area of investigation. As part of our culturomics project that consists of exploring the human microbiota by isolating bacteria through innovative culture-dependent methods, we isolated a new bacterial strain from the back of the right hand, in a 67-year-old healthy woman. Here, we characterize the strain Marseille-Q2903 by the taxonogenomic approach. Marseille-Q2903 exhibits a 99.5% 16S rRNA sequence similarity with *Brachybacterium muris*^T^ but with only 92% of coverage. The closest species based on a 100% coverage of the 16S sequence is *Brachybacterium timonense*^T^ with an identity similarity of 97.63%. Furthermore, digital DNA-DNA hybridization reveals a maximum identity similarity of only 31.5% and an OrthoANI parameter provided a value of 86.95% between Marseille-Q2903 and *Brachybacterium muris*^T^. Marseille-Q2903 is a yellowish-pigmented, Gram-positive, coccoid shaped, and facultative aerobic bacterium, and belonging to the Dermabacteraceae family. The major fatty acids detected are 12-methyl-tetradecanoic acid (69%), 14-methyl-hexadecanoic acid (16%), and 14-methyl-pentadecanoic acid (7%). Marseille-Q2903 genome size is of 3,073,790 bp, with a 70.43% G + C content. Taken altogether, these results confirm the status of this strain as a new member of the *Brachybacterium* genus for which the name of *Brachybacterium epidermidis* sp. strain Marseille-Q2903^T^ is proposed (=CSURQ2903^T^ = CECT30363).

## 1. Introduction

For several decades, with the improvement of molecular tools for bacterial identification, culture has been neglected in favor of metagenomics and 16S rRNA pyrosequencing. Since the 2010s, the design of new culture conditions has returned to the forefront thanks to the development of the culturomics method, which is based on the diversification of culture conditions. By selection of different compounds, this can lead to mimicking the natural environment, or to unhide the minority species through selection processes [1, 2]. For instance, the use of antibiotics has allowed to culture previously underestimated Gram-negative bacteria isolated from human skin [3]. The beneficial and protective role of bacterial communities in close relationship with the skin is at a turning point. The ensued findings will certainly allow its clinical manipulation and will also be an important springboard for industrial concern through the investigation of microbial-derivated products with bioactive activities [4].

The isolation of *Brachybacterium epidermidis* strain Marseille-Q2903 arise as part of the culturomics project declined to the exploration of the skin microbiota. This bacterium was initially isolated from the back of the hand of a 67-year-old healthy woman. Here, we describe this new bacterial species, *Brachybacterium epidermidis* strain Marseille-Q2903 using the taxonogenomics polyphasic approach, including phenotypic characterization, wall fatty acid composition, and phylogenomic analyses.

## 2. Materials and Methods

### 2.1. Sample Acquisition and Strain Isolation

The sample was obtained by swabbing a 10 cm^2^ area of the skin from the right hand of a 67-year-old healthy woman. The study was validated by the Ethics Committee Sud-Est IV under the ID-RCB: 2019-A01508-49. Informed consent was obtained from the volunteers. After being mixed with the transport media, the skin sample was diluted to 1 : 100 in PBS (Dulbecco's phosphate buffered saline, Sigma-Aldrich), and 50 *μ*L of each dilution was directly seeded in Columbia agar (bioMérieux, Marcy-l'Etoile, France) or homemade R2A plates (all components obtained from Sigma-Alrich), incubated under aerobic conditions at 31°C. Plates were visualized every day until five days and subcultures were seeded in another Columbia agar plate maintained 24 hours under aerobic conditions at 31°C. To identify the strain Marseille-Q2903, a MALDI-TOF mass spectrometry (MS) protein analysis was carried out in triplicate using a Microflex spectrometer (Bruker Daltonics, Bremen, Germany) but failed, suggesting that the generated spectra were not in the database. Strain spectra were imported into the MALDI BioTyper software (version 3.0, Bruker, Bremen, Germany) and analyzed by standard pattern matching with default parameters. Our database (https://www.mediterranee-infection.com/access-resources/base-de-donnees/urms-data-base/) was then incremented with the spectrum of this new bacterial species.

### 2.2. Phenotypic Tests

Different growth temperatures (20°C, 31.5°C, 37°C, 45°C, and 56°C), atmosphere conditions (anaerobic, aerobic, and microaerophilic) using generator bags (CampyGEN, Oxoid, USA) and pH conditions (5, 6.5, 7.5, and 8.5) were tested. Plates were prepared by using Columbia agar base powder (Sigma-Aldrich). Biochemical properties of these strains were tested using API ZYM, API 20 NE, API 20E, and API 50 CH strips (bioMérieux, Marcy L'Étoile, France) according to the manufacturer's instructions. Catalase and oxydase activity were respectively evaluated with ID-ASE (Biomérieux SA, Marcy-l'Etoile, France) and the contact test with H_2_O_2_ (Sigma-Aldrich). To evaluate the bacterial structure, a colony was collected from agar and immersed into a 2.5% glutaraldehyde fixative solution. The suspension was vortexed, passed ten times through a 21-gauge needle to separate bacterial colonies, and fixed on an uncoated glass slide by cytocentrifugation. A 1% ammonium molybdate-negative stain was applied for 1 minute before gently washing the slide with 0.2 *μ*m-filtered distilled water. The slide was air-dried and examined by scanning electron microscopy on a TM4000 microscope (Hitachi High-Tech, HHT, Tokyo, Japan) with a 15 kV voltage. Motility test was performed using the semisolid TCC media as described by Tittsler and Sandholzer [5]. Sporulation was evaluated by collecting a colony from agar in 1 mL of phosphate buffer saline (PBS) and using a thermal shock for 10 minutes at 80°C. Cellular fatty acid methyl ester (FAME) analysis was performed by GC/MS. Two samples of strain Marseille-Q2903 were prepared with approximately 110 mg of bacterial biomass per tube harvested from several culture plates. Fatty acid methyl esters were prepared as described by Sasser [6]. GC/MS analyses were carried out as described before [7]. Briefly, fatty acid methyl esters were separated using an Elite 5-MS column and monitored by mass spectrometry (Clarus 500-SQ, 8S, Perkin Elmer, Courtaboeuf, France). Spectral database search was performed using MS Search 2.0 operated on the Standard Reference Database 1A (NIST, Gaithersburg, USA) and the FAMEs mass spectral database (Wiley, Chichester, UK).

### 2.3. Genome Sequencing, Annotation, and Genome Comparison

Genomic DNA (gDNA) of strain Marseille-Q2903 was extracted in two steps: a mechanical treatment was first performed by glass beads acid washed (G4649-500g Sigma) using a FastPrep-24™ 5G Grinder (mpBio) at maximum speed (6.5) for 90 s. Then, after 30 minutes lysozyme incubation at 37°C, DNA was extracted using the EZ1 biorobot (Qiagen) with the EZ1 DNA tissue kit. The elution volume was of 50 *μ*L. gDNA was quantified by a Qubit assay with the high sensitivity kit (Life technologies, Carlsbad, CA, USA) to 0.2 ng/*μ*l. Genomic DNA was next sequenced using the MiSeq Technology (Illumina Inc, San Diego, CA, USA) with the paired end strategy prepared with the Nextera XT DNA sample prep kit (Illumina). To prepare the paired end library, a dilution was performed to require 1 ng of the genome as input to prepare the paired end library. The «tagmentation» step fragmented and tagged the DNA. Then, limited cycle PCR amplification (12 cycles) completed the tag adapters and introduced dual-index barcodes. After purification on AMPure XP beads (Beckman Coulter Inc, Fullerton, CA, USA), the libraries were then normalized on specific beads according to the Nextera XT protocol (Illumina). Normalized libraries were pooled into a single library for sequencing on the MiSeq. The pooled single-strand library was loaded onto the reagent cartridge and then onto the instrument along with the flow cell. To improve the quality of the assemblies, an Oxford Nanopore approach was performed on 1D genomic DNA sequencing using the MinIon device using the SQK-LSK109 kit. Library was constructed from 1 *μ*g genomic DNA without fragmentation and end repair. Adapters were ligated to both ends of genomic DNA. After purification on AMPure XP beads (Beckman Coulter Inc, Fullerton, CA, USA), the library was quantified by a Qubit assay with the high sensitivity kit (Life technologies, Carlsbad, CA, USA). The workflow WIMP was chosen for bioinformatic analysis in live.

Genome annotation was obtained through the NCBI prokaryotic genome annotation pipeline [8]. The genome sequence data were uploaded to the Type (Strain) Genome Server (TYGS), a free bioinformatics platform available under https://tygs.dsmz.de, for whole genome-based taxonomic analysis [9]. Determination of the closest type strain genomes was conducted in two complementary ways: first, all user genomes were compared against all type strain genomes available in the TYGS database via the MASH algorithm, a fast approximation of intergenomic relatedness, [10] and the ten type strains with the smallest MASH distances chosen per user genome. Second, an additional set of ten closely related strains was determined via the 16S rDNA gene sequences. These were extracted from the user genomes using RNAmmer [11], and each sequence was subsequently BLASTed [12] against the 16S rDNA gene sequence of each of the currently 12983 strains available in the TYGS database. This was used as a proxy to find the best 50 matching strains (according to the bitscore) for each user genome and to subsequently calculate precise distances using the Genome BLAST Distance Phylogeny (GBDP) approach under the algorithm “coverage” and distance formula d5. [13]. These distances were finally used to determine the 10 closest type strain genomes for each of the user genomes. All pairwise comparisons among the set of genomes were conducted using GBDP and accurate intergenomic distances inferred under the algorithm “trimming” and distance formula d5. 100 distance replicates were calculated each. Digital DDH values and confidence intervals were calculated using the recommended settings of the GGDC2. Complementarily, the degree of genomic similarity of interest strains with closely related species was estimated using the orthologous average nucleotide identity (OrthoANI) software with default parameters, [14] the closest species were determined with the DDH basis. Trees were inferred with FastME 2.1.6.1 [15] from GBDP distances calculated from 16S rDNA gene sequences or whole-genome sequence. The branch lengths are scaled in terms of GBDP distance formula d5. The numbers above branches are GBDP pseudobootstrap support values >60% from 100 replications, with an average branch support of 84.3%. The tree was rooted at the midpoint and regenerated with the iTOL Tool v5. [16]. Antibiotic resistance genes and presence of pathogenesis-related proteins were investigated using the ABRicate tools v1.0.1 against ARG-ANNOT [17], EcOH [18], NCBI Bacterial Antimicrobial Resistance Reference Gene Database [19], PlasmidFinder [20], ResFinder [21], CARD [22], and VFDB [23] using the Online Galaxy platform. [24].

## 3. Results

### 3.1. Strain Marseille-Q2903 Identification

**Figure 1:**
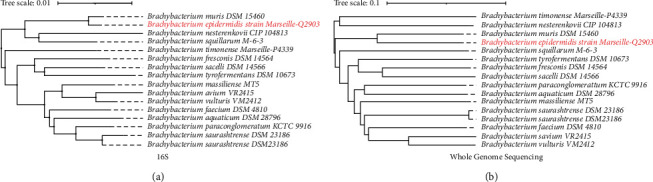
(a) 16s rRNA-based phylogenetic tree. (b) Whole genome-based phylogenetic tree highlighting the position of *B*. *epidermidis* sp nov. strain Marseille-Q2903^T^, relative to other closely related bacterial species.

**Figure 2:**
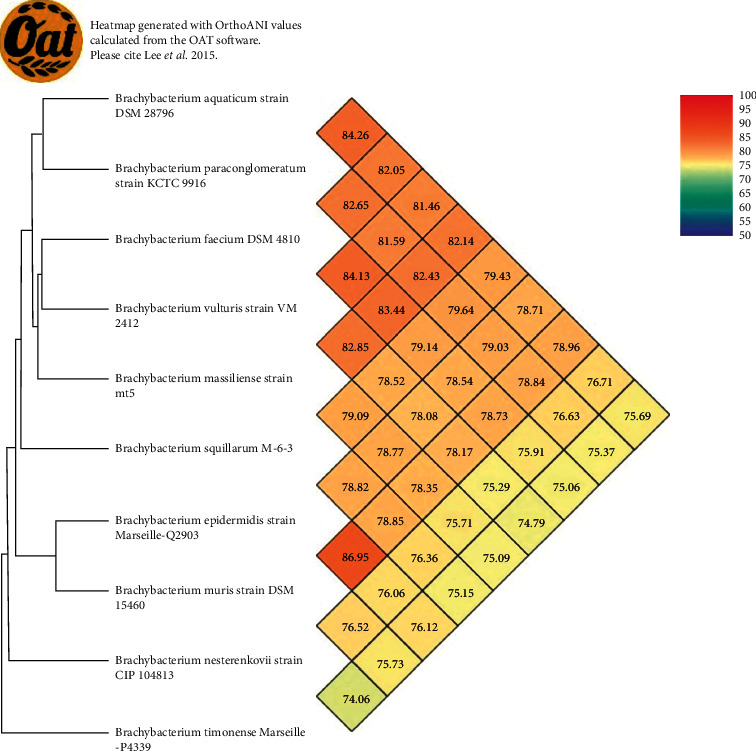
Heatmap generated with orthologous average nucleotide identity (OrthoANI) values calculated using the OAT software, comparing strain Marseille-Q2903T with other closely related bacterial species.

Strain Marseille-Q2903 exhibited a 99.5% 16S rRNA sequence similarity with *Brachybacterium muris*^T^ (accession number: NR_024571.1) but with 92% of coverage (Figure 1(a)). The closest species based on a 100% coverage of the 16S rRNA sequence is *Brachybacterium timonense*^T^ with a sequence similarity of 97.63% (accession number LT962482.1). Furthermore, digital DNA-DNA hybridization revealed an identity percentage of 31.5% (Table S1). OrthoANI parameter provided a value of 86.95% (Figure 2) between the new bacterial strain and *Brachybacterium muris*^T^. Taken altogether, these results confirm the status of this strain as a new member of the *Brachybacterium* genus for which the name of *Brachybacterium epidermidis* Marseille-Q2903T is proposed.

### 3.2. Phenotypic Characteristics of *Brachybacterium epidermidis* Strain Marseille-Q2903

**Figure 3:**
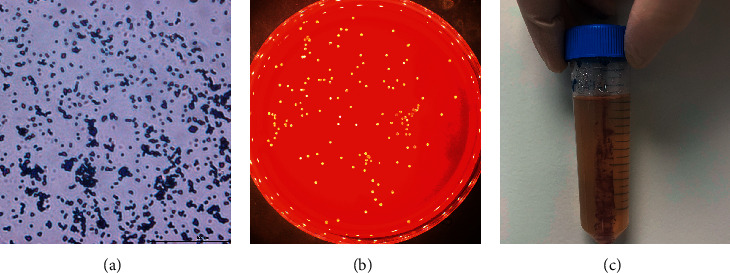
Phenotypic characteristics of *Brachybacterium epidermidis* strain Marseille-Q2903^T^. (a) Gram staining; (b) visualization of the colonies; and (c) motility test.

**Figure 4:**
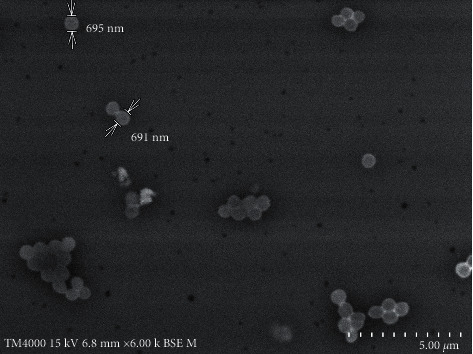
Scanning electron microscopy of *B*. *epidermidis* sp. nov. strain Marseille-Q2903^T^ using a TM4000 microscope (Hitachi High-Tech, HHT, Tokyo, Japan).

*Brachybacterium epidermidis* strain Marseille-Q2903 was a facultatively anaerobic bacterium that grew on 5% sheep blood agar. This Gram-positive bacterium formed small yellow colonies and did not hemolyze (Figure 3). Its shape was coccoid with a size of about 0.6-0.7 *μ*m (Figure 4). It was nonmotile and did not sporulate. The optimum temperature for the growth of this bacterium was between 31.5 and 37°C. The optimal pH for its growth was of 8.5.

Most of the fatty acids found in *Brachybacterium epidermidis* were branched structures (Table S2). These were 12-methyl-tetradecanoic acid (69%), 14-methyl-hexadecanoic acid (16%), and 14-methyl-pentadecanoic acid (7%). Unsaturated fatty acids were detected in smaller quantities. API, ZYM, 20 NE, and 50CH galleries were performed, and the positive reactions for enzymes were as follows: esterase (C4), esterase lipase (C8), leucine arylamidase, valine arylamidase, naphthol-AS-BI phosphohydrolase, *β*-galactosidase, *α*-glucosidase, N-acetyl-*β*-glucosaminidase, *α*-mannosidase, glycerol, D-ribose, ferric esculin citrate, D-maltose, D-sucrose, D-trehalose, starch, glycogen, D-melezitose, 4-nitrophenyl-*β*D-galactopyranoside, and sodium pyruvate. Other reactions in the API galleries were negative. For *Brachybacterium epidermidis*, the oxidase test was negative, and the catalase test was positive. Phenotypic differences that discriminate *Brachybacterium epidermidis* from its closest relatives were the majority found through its metabolism characteristics (Table 1) [25–28]. Among others, production of *α*-glucosidase was positive for *B*. *epidermidis* while it was negative for *B*. *squillarum*. Production of *β*-galactosidase was positive for *B*. *epidermidis* while it was negative for *B*. *paraconglomeratum* and *B*. *squillarum*.

### 3.3. Genome Analysis of *Brachybacterium epidermidis* Strain Marseille-Q2903

**Figure 5:**
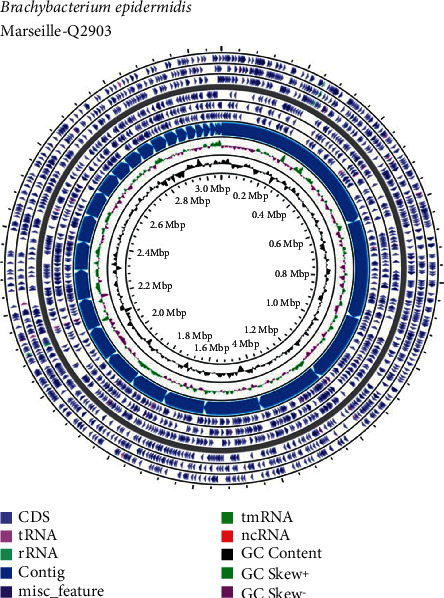
Graphical circular map of the genome of *B*. *epidermidis* strain Marseille-Q2903^T^ obtained by using the CGView server.

The genome size of strain Marseille-Q2903 was 3,073,790-bp long with a 70.43% G + C content. The genome assembly of this strain was achieved with 31 contigs (with 7.0*x* coverage). Of the 2,805 predicted genes, 2,587 were protein-coding genes and 58 were RNAs (2 16S rRNA, 2 5S rRNAs, 2 23S rRNAs, 49 tRNAs, and 3 ncRNAs) (Figure 5).

The in silico resistome of the strain Marseille-Q2903T and the search for virulence factors of this strain showed resistance genes and neither virulence factor genes. Distribution of functional classes of predicted genes according to the clusters of orthologous groups of proteins showed that the genome of *Brachybacterium epidermidis* showed a coherent structure compared to their closely related species (Figure S1).

## 4. Discussion

As regards the strain Marseille-Q2903, both phylogenetic and phenotypic analysis revealed several different characteristics when compared to other members of the Dermabacteraceae family, suggesting a classification as a new species of the *Brahcybacterium* genus.

The Dermabacteriaceae family includes 4 genera, *Helcobacillus*, *Dermabacter*, *Devriesea* (these three latter are all monospecific), and *Brachybacterium* that includes 23 validly published species [29]. The first representant of this genus was isolated in 1966 from a poultry deep litter within others bacteria [30] but that is only in 1988 that the type species was classified and named due to the advance in molecular biology [31].

The genomic content, through dDDH and OrthoANI values, of strain Marseille-Q2903 (31.5% and 86.95, respectively) comforted its new species status. Indeed, a DDH value equal to or higher than 70% has been recommended as a suitable threshold for the definition of members of a species, and approximately 95–96% average nucleotide identity values are considered as the species boundary [14, 32]. Therefore, we propose Marseille-Q2903 as the type strain of a new species within the *Brachybacterium* genus under the name of *Brachybacterium epidermidis*, *Gr*. *masc*. *adj*. (*βραχύς*) *brachys*, *short*; *N*.*L*. *neut*. *n*. *bacterium*, *a rod*; *N*.*L*. *neut*. *n*. *Brachybacterium*, *a small rod*. *epidermidis*: e.pi.der'mi.dis Gr. neut. n. (*ἐπίδερμα*) *epiderma*, the outer skin; N.L. gen. n. *epidermidis*, of the epidermis.

## Figures and Tables

**Table 1:** Differential phenotypic characteristics of *Brachybacterium epidermidis* strain Marseille-Q2903^T^ and closely related bacterial species.

	*B*. *epidermidis*	*B*. *paraconglomeratum*	*B*. *massiliense*	*B*. *squillarum*	*B*. *faecium*	*B*. *saurashtrene*
	Marseille-Q2903	KCTC 9916	MT5	M-6-3	DSM 4810	DSM23186
Properties						
Cell diameter (*μ*m)	0.6–0.7 *μ*m	0.5 to 1 *μ*m	0.5 to 0.9 *μ*m	1.0 to 1.5 *μ*m	0.5–0.75 × 1.5–2.5 *μ*m	0.3–0.75 *μ*m
Oxygen requirement	Facultative	Facultative	+	+	Facultative	+
Gram strain	+	+	+	+	+	+
Motility	−	−	−	−	−	−
Endospore formation	−	−	−	−	−	NA
Optimum temperature for growth (°C)	31.5–37°C	NA	37°C	45°C	25–30°C	30°C
Production of						
Alkaline phosphatase	−	NA	−	NA	NA	NA
Catalase	+	+	+	−	+	+
Oxidase	−	−	−	−	−	−
*α*-Glucosidase	+	NA	+	−	NA	NA
*β*-Galactosidase	+	NA	−	−	NA	NA
Acid from						
N-Acetylglucosamine	+	NA	−	−	NA	NA
L-Arabinose	−	+	−	−	+	−
D-Ribose	+	−	+	−	+	−
D-Mannose	−	+	−	+	+	+
D-Mannitol	−	−	+	+	+	NA
D-Glucose	+	+	+	−	+	+
D-Fructose	−	+	+	−	−	+
D-Maltose	+	+	+	+	+	+
D-Lactose	−	+	+	−	−	+
G + C content (mol%)	70.43	68.6	NA	71.5	72.05	73
Isolation sources	Human healthy skin	Obtained from corn steep liquor	Stool from a healthy Senegalese child	Salt-fermented seafood	Deep litter (soil)	*Salicornia* plants

## Data Availability

*Brachybacterium epidermidis* strain Marseille-Q2903T was deposited in CSUR collection under accession CSUR-Q2903 and in CECT collection under number CECT30363. The 16S rRNA and genome sequences are available on GenBank under accession numbers MW186831 and JADEYR000000000.1, respectively.

## Supplementary Materials

Supplementary Materials Table S1: digital DNA-DNA hybridization values obtained by sequence comparison of all studied genomes using TYGS second value. Table S2: cellular fatty acid composition (%) of *Brachybacterium epidermidis* strain Marseille-Q2903^T^. Figure S1: distribution of functional classes of predicted genes according to the clusters of orthologous groups of proteins of *Brachybacterium epidermidis* strain Marseille-Q2903^T^ and its closely related bacterial species.
