# Supplementary material for: Brachybacterium epidermidis Sp. Nov., a Novel Bacterial Species Isolated from the Back of the Right Hand, in a 67-Year-Old Healthy Woman
Source: Int J Microbiol. 2022 Mar 29;2022:2875994. doi: 10.1155/2022/2875994 (PMC8983266; doi:10.1155/2022/2875994)
Supplement: Supplementary Materials — Table S1: digital DNA-DNA hybridization values obtained by sequence comparison of all studied genomes using TYGS second value. Table S2: cellular fatty acid composition (%) of Brachybacterium epidermidis strain Marseille-Q2903T. Figure S1: distribution of functional classes of predicted genes according to the clusters of orthologous groups of proteins of Brachybacterium epidermidis strain Marseille-Q2903T and its closely related bacterial species. [file 2875994.f1.zip › 2875994.f1/TableS1 (1).pdf]

	dDDH with *Brachybacterium epidermidis* strain Q2903 (in %)	C.I. (in %)	G+C content difference (in %)
Brachybacterium muris DSM 15460	31,5	[29,1 - 34]	0,7
Brachybacterium paraconglomeratum KCTC 9916	22,7	[20.4 - 25.2]	1,8
Brachybacterium aquaticum DSM 28796	22,6	[20.3 - 25.1]	1,61
Brachybacterium massiliense MT5	22,3	[20.0 - 24.8]	0,22
Brachybacterium squillarum M-6-3	22,1	[19.9 - 24.6]	2,35
Brachybacterium faecium DSM 4810	22	[19.7 - 24.4]	1,62
Brachybacterium saurashtrense DSM23186	21,7	[19.5 - 24.2]	2,32
Brachybacterium timonense Marseille-P4339	21,6	[19.3 - 24.0]	3,14
Brachybacterium vulturis VM2412	21,5	[19.3 - 24.0]	0,41
Brachybacterium nesterenkovii CIP 104813	21,5	[19.2 - 23.9]	2
Brachybacterium ginsengisoli KCTC 29226	21,4	[19.1 - 23.8]	1,14
Brachybacterium avium VR2415	21,3	[19.1 - 23.8]	0
Brachybacterium endophyticum M1HQ-2	20,6	[18.4 - 23.0]	0,62
