# Supplementary material for: Brachybacterium epidermidis Sp. Nov., a Novel Bacterial Species Isolated from the Back of the Right Hand, in a 67-Year-Old Healthy Woman
Source: Int J Microbiol. 2022 Mar 29;2022:2875994. doi: 10.1155/2022/2875994 (PMC8983266; doi:10.1155/2022/2875994)
Supplement: Supplementary Materials — Table S1: digital DNA-DNA hybridization values obtained by sequence comparison of all studied genomes using TYGS second value. Table S2: cellular fatty acid composition (%) of Brachybacterium epidermidis strain Marseille-Q2903T. Figure S1: distribution of functional classes of predicted genes according to the clusters of orthologous groups of proteins of Brachybacterium epidermidis strain Marseille-Q2903T and its closely related bacterial species. [file 2875994.f1.zip › 2875994.f1/TableS2 (1).pdf]

Fatty acids	Name	Mean relative % [a]
15:0 anteiso	12-methyl-tetradecanoic acid	69.2 ± 5.0
17:0 anteiso	14-methyl-Hexadecanoic acid	15.9 ± 3.0
16:0 iso	14-methyl-Pentadecanoic acid	6.6 ± 1.1
18:1n7	11-Octadecenoic acid	2.8 ± 0.3
15:0 iso	13-methyl-tetradecanoic acid	1.5 ± 0.7
14:0 iso	12-methyl-Tridecanoic acid	1.3 ± 0.3
16:0	Hexadecanoic acid	1.3 ± 0.3
18:2n6	9,12-Octadecadienoic acid	TR
18:1n9	9-Octadecenoic acid	TR
17:0 iso	15-methyl-Hexadecanoic acid	TR

[a] Mean peak area percentage ; TR = trace amounts < 1 %
