# Supplementary figures and images for: Brachybacterium epidermidis Sp. Nov., a Novel Bacterial Species Isolated from the Back of the Right Hand, in a 67-Year-Old Healthy Woman
Source: Int J Microbiol. 2022 Mar 29;2022:2875994. doi: 10.1155/2022/2875994 (PMC8983266; doi:10.1155/2022/2875994)

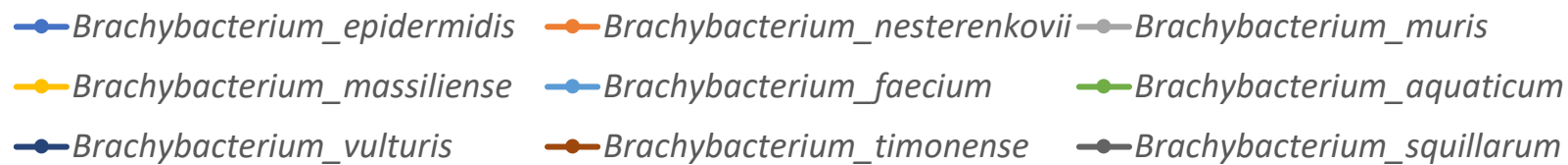

Supplement: Supplementary Materials — Table S1: digital DNA-DNA hybridization values obtained by sequence comparison of all studied genomes using TYGS second value. Table S2: cellular fatty acid composition (%) of Brachybacterium epidermidis strain Marseille-Q2903T. Figure S1: distribution of functional classes of predicted genes according to the clusters of orthologous groups of proteins of Brachybacterium epidermidis strain Marseille-Q2903T and its closely related bacterial species. [file 2875994.f1.zip › 2875994.f1/FigS1 (1).pdf]
